# The Human CD8β M-4 Isoform Dominant in Effector Memory T Cells Has Distinct Cytoplasmic Motifs That Confer Unique Properties

**DOI:** 10.1371/journal.pone.0059374

**Published:** 2013-03-22

**Authors:** Deepshi Thakral, Maria M. Coman, Arunima Bandyopadhyay, Sunil Martin, James L. Riley, Paula B. Kavathas

**Affiliations:** 1 Departments of Laboratory Medicine and Immunobiology, Yale University School of Medicine, New Haven, Connecticut, United States of America; 2 Abramson Family Cancer Research Institute and Department of Pathology and Laboratory Medicine, University of Pennsylvania School of Medicine, Philadelphia, Pennsylvania, United States of America; New York University, United States of America

## Abstract

The CD8 co-receptor influences T cell recognition and responses in both anti-tumor and anti-viral immunity. During evolution in the ancestor of humans and chimpanzees, the CD8B gene acquired two additional exons. As a result, in humans, there are four CD8β splice variants (M1 to M4) that differ in their cytoplasmic tails. The M-1 isoform which is the equivalent of murine CD8β, is predominantly expressed in naïve T cells, whereas, the M-4 isoform is predominantly expressed in effector memory T cells. The characteristics of the M-4 isoform conferred by its unique 36 amino acid cytoplasmic tail are not known. In this study, we identified a dihydrophobic leucine-based receptor internalization motif in the cytoplasmic tail of M-4 that regulated its cell surface expression and downregulation after activation. Further the M-4 cytoplasmic tail was able to associate with ubiquitinated targets in 293T cells and mutations in the amino acids NPW, a potential EH domain binding site, either enhanced or inhibited the interaction. In addition, the M-4 tail was itself mono-ubiquitinated on a lysine residue in both 293T cells and a human T cell line. When peripheral blood human T cells expressed CD8αβ M-4, the frequency of MIP-1β secreting cells responding to antigen presenting cells was two-fold higher as compared to CD8αβ M-1 expressing T cells. Thus, the cytoplasmic tail of the CD8β M-4 isoform has unique characteristics, which likely contributed to its selective expression and function in human effector memory T cells.

## Introduction

Human T cells are categorized into subsets based on stage of differentiation and lineage. The cytotoxic CD8 T lymphocyte (CTL) plays a primary role in protection against cells infected by intracellular pathogens and transformed tumor cells [1]. CD8 functions as a co-receptor with the T cell receptor (TCR) by simultaneous binding to a major histocompatibility complex I (MHCI) protein where the TCR contacts peptide+MHCI and CD8 binds to a site that is relatively less polymorphic. CD8 plays a critical role in distinguishing antigen quality and in T cell receptor activation [2]. For instance, the CD8αβ co-receptor enhanced TCR sensitivity for pMHCI by at least one million-fold when TCR-pMHCI affinities were in the physiological range [3]. CD8 facilitates signal transduction by delivering p56*^lck^* kinase to the CD3-TCR complex resulting in phosphorylation of tyrosines on CD3ζ [4] and on the recruited adaptor protein ZAP-70 kinase [5]. This leads to recruitment of the scaffold protein LAT (linker of activated T cells) and its associated proteins such as Grb-2 and Sos1 [6], [7] as part of a signaling cascade controlling T cell activation. The p56*^lck^* kinase also phosphorylates the clathrin H chain, a regulatory step in endocytosis of the TCR and CD8 [8].

The human CD8 protein has an alpha and beta subunit that can form αα, αβ or ββ dimers. While the CD8α chain associates with p56*^lck^* kinase, the CD8β chain plays an important role in T cell function [3], [9], [10]. The N-terminal immunoglobulin (Ig)-like domain and a palmitoylation site in the cytoplasmic tail of CD8β chain contributes to partitioning of CD8 into the plasma membrane lipid rafts where signaling proteins such as p56*^lck^* are enriched [11], [12]. The CD8β chain enhances association of CD8αβ with p56*^lck^* and LAT as compared with CD8αα [13], [14]. A direct association of CD8β with the CD3δ-chain of the TCR-CD3 complex was reported which contributes to recruitment of the TCR into lipid rafts [15]. Following CD3 engagement, the selective co-internalization of the TCR with CD8αβ but not with CD8αα affects CTL activity [9], [16]–[18] indicating a functional role for CD8β in this process.

The evolution of the CD8β gene further supports the importance of this protein. Genes of the immune system show a relatively rapid evolution and this includes the CD8B gene [19] that acquired two additional exons in the human and chimpanzee lineage but not rhesus macaque. In humans the transcribed mRNA undergoes alternative splicing giving rise to four different membrane-associated isoforms (M-1, M-2, M-3 and M-4) where M-1 is the murine counterpart [20], [21]. The human CD8β M-1 to M-4 isoforms share a common extracellular, transmembrane and membrane-proximal cytoplasmic sequence, which contains the palmitoylation site, while the remaining cytoplasmic tails have either 3, 39, 14, or 36 unique amino acids, respectively (Figure S1). These isoforms showed differential mRNA expression patterns in peripheral human CD8 T cells. We reported that naïve and central memory CD8 T cells expressed M-1>M-4>M-2 while in effector memory T cells the mRNA for the M-4 isoform was predominant [22].

In this study, we focused on characterizing the properties of the M-4 isoform to better understand the functional advantage that this isoform might confer to the CD8 coreceptor in effector memory T cells. Memory T cells have a lower threshold of activation, are long-lived, and are broadly classified into either effector or central memory cells [23]. In general, effector memory T cells preferentially home to peripheral tissues, are poised for immediate effector functions but have reduced proliferative capacity. The profile of cytokines that are secreted by T cells can vary depending on the stage of differentiation. The polarization of late effector memory CD8 T cells toward CC chemokine production such as MIP-1β and decreased IL-2 production suggests a unique functional role for this subset [24].

We found distinct differences between the M-4 isoform as compared with the M-1 isoform. We identified unique motifs in the cytoplasmic tail of M-4 isoform that regulated its cell surface expression, down-regulation, and interaction with ubiquitinated proteins. The protein could itself be post-translationally modified by ubiquitination of lysine residues. In a functional assay measuring cytokine expression after activation, we found increased frequency of MIP-1β responders when T cells expressed the M-4 isoform as compared with the M-1 isoform. These unique properties of the M-4 isoform distinctly influence the CD8 coreceptor function and most likely contribute to the properties of effector memory T cells in human immunity.

## Materials and Methods

### Ethics Statement

Human cells were obtained under protocol (#8706003674) approved by Yale Human Investigation Committee. Written informed consent was obtained from every donor.

### Plasmid Constructs, Site-directed Mutagenesis

Lentiviral vectors (pELNS) were constructed by inserting CD8β isoforms M-1 to M-4 into the AvrII and SalI restriction sites downstream of GFP [25]. Mutagenesis of pcDNA3.1 vector (Invitrogen, Carlsbad, CA) carrying CD8β M-4 was performed with QuickChange mutagenesis kit using oligos listed in Table S1. Mutants were then sub-cloned into the pELNS vector containing the cDNA encoding codon optimized CD8α upstream (717 bp synthesized with NheI and BspEI at the 5′ and 3′ end respectively).

### Antibodies and Reagents

Antibodies used include: anti-CD8β 5F2 (Santa Cruz, Santa Cruz, CA) or SIDIBEE (eBioscience, San Diego, CA), anti-CD8αβ 2ST8.5H7 (Beckman Coulter Inc., Brea, CA), anti-CD8α OKT8 (ATCC hybridoma, FITC labeled), Alexa-Fluor647 conjugated anti-CD3, anti-GAPDH (Sigma-Aldrich, St. Louis, MO), anti-HA (6E2) (Cell Signaling, Danvers, MA), and anti-Ub (P4D1, Biolegend, San Diego, CA). Secondary antibodies include goat anti-mouse PE or APC (Jackson Laboratories, Bar Harbor, ME) and goat anti-mouse HRP (eBioscience, San Diego, CA). The HLA-A2 tetramer with the NY-ESO-1 peptide (SLLMWITQV) was provided by the NIH tetramer facility.

### Multicolor Intracellular Cytokine Staining Assay

For each test, 5×10^4^ T cells were cultured with 2.5×10^5^ targets in 200 µl of T cell growth medium (AIMV with 3% human serum) in a 96-well U-bottom plate and incubated for 1 hr. Cells were incubated for 5–6 hrs with 5 µg/ml of Golgi stop (BD Biosciences, San Jose, CA) and 5 µg/ml of Golgi plug (BD Biosciences, San Jose, CA). After incubation, EDTA was added to a final concentration of 2 mM and cells were washed with PBS. They were then incubated for 30 min with anti-CD8-PE-Cy5 (Biolegend, San Diego, CA) and violet amine reactive viability dye (Invitrogen, Carlsbad, CA). After two washes with PBS, the cells were fixed and permeabilized in 200 µl of Cytofix/Cytoperm solution (BD Biosciences, San Jose, CA) for 20 min at RT, followed by 2 washes with Perm/Wash buffer (BD Biosciences, San Jose, CA). Final staining was with anti-IL-2-allophycocyanin (Biolegend, San Diego, CA), anti-IFN-γ Alexa Fluor 700 (Biolegend, San Diego, CA), anti-MIP-1β-PE (BD Biosciences, San Jose, CA) and streptavidin-PE-Texas red (BD Biosciences, San Jose, CA) for 30 min at RT. Samples were washed 4 times with BD Perm/Wash buffer, fixed with Cytofix Cytoperm (Becton Dickenson, Franklin Lakes, NJ) for 20 min at RT, washed and stored at 4°C overnight. Approximately 10,000 CD8^+^ events per sample were acquired on an LSRII instrument (BD Biosciences, San Jose, CA) and data were processed with FlowJo software (Tree Star Inc., San Carlos, CA).

### Cell Lines and Peripheral Blood lymphocytes (PBLs)

HEK-293T human embryonic kidney cells from American Type Culture Collection (Manassas, VA), JM Thymoma (CD4^+^, CD8α^+^, CD8β^−^) [26] and H9 lymphoblast T cell lines (CD4^+^, CD8^−^) [27] were cultured in RPMI 1640 supplemented with 10% FBS, 2 mM glutamine, 100 IU/ml penicillin, and 100 µg/ml streptomycin. PBLs were isolated as described [22]. Primary CD4^+^ T cells were purified by negative isolation of CD4^+^ T cells using Dynabeads (Invitrogen, Carlsbad, CA) yielding 90–95% purity.

### Production of High Titer Lentiviral Vectors and Transduction into CD4^+^ T Cells

Lentiviral vectors were produced by transfection of HEK293T cells as described earlier [28].Viral supernatant was harvested at 48 hrs post-transfection and virus was concentrated with PEG-It virus precipitation solution (System Biosciences, Mountain View, CA). Virus titer was determined in H9 cells and analysis of protein expression by antibody binding and flow cytometry. Purified CD4^+^ T cells were stimulated by adding anti-CD3/CD28 beads (1∶1 bead:cell ratio) (Invitrogen, Carlsbad, CA) and seeded into a 48-well plate at 10^6^ cells/mL in 250 µl T cell growth medium (AIMV with 3% human serum and 300 U/ml recombinant IL-2). After 24 hours roughly 250 µl of virus was added and next day the medium was changed by replacing 250 µl with fresh medium supplemented with IL-2. On day 5, culture was debeaded, supernatant was removed and pellet was resuspended in 500 µl of AIMV medium supplemented with IL-2. From day 8, cells were counted and the day they stopped expanding, restimulated with antigen presenting cells.

### FACS Analysis and Sorting

Single or multi-color immunostaining was performed using standard protocol. Cells were gated on forward/side scatter and then for similar GFP levels, sorted using BD FACSAria cell sorter, and data processed with FlowJo software.

### Receptor Downregulation Assays

Cells were stained with anti-CD8 antibody (as indicated) for 30 min on ice, washed and resuspended in media with or without PMA (100 ng/ml) and incubated at 37°C for 1 hr. Cells were washed with cold PBS containing 1% FBS and 1% azide to block downregulation and stained with PE conjugated goat anti-mouse IgG to determine levels of CD8 remaining on the cell surface. After extensive washing anti-CD3 conjugated to Alexa Fluor 647 (Biolegend, San Diego, CA) was added for 20 minutes on ice. Cells were then washed and analyzed on a flow cytometer. Percentage of receptor downregulation was calculated using the mean fluorescence intensities and the following equation: % downregulation = [1-MFI CD8β (stimulated)/MFI CD8β (unstimulated)]×100.

### Cell Lysis, Immunoprecipitation, SDS-PAGE, and Western Blotting

Cells were solubilized in lysis buffer [50 mM Tris-HCl pH 7.6, 140 mM NaCl, 2 mM EDTA and one of the following detergents: 1% IGEPAL CA-630, 1% BRIJ-97, or 1% SDS and 1% Triton-X100] supplemented with protease inhibitor cocktail of NaF, leupeptin, and PMSF (1 mM). For ubiquitination analysis the following blockers were added: 20 mM N-Ethylmaleimide and 10 mM 1,10-phenatholine monohydrate (Sigma-Aldrich, St. Louis, MO), with 50 uM PR-619 (LifeSensors, Malvern, PA). Immunoprecipitation with respective antibodies and Western blotting protocols were previously described [22]. The CD8 protein was immunoprecipitated with the anti-CD8β antibody (SIDIBEE) from eBioscience (San Diego, BA) that was more efficient than the 5F2 antibody. The 5F2 antibody was used for Western blotting. The signal intensity of a band on the Western blots was quantitated using the BioRad program Quantity One Software.

### Assay for Cytokine/Chemokine Secretion

Cytokine/chemokine production was determined by multiplex technology after stimulation of JM thymoma. Antibody cross-linking with plate bound anti-CD3 and anti-CD28 (Biolegend, San Diego, CA) was used to stimulate each JM cell-line for 24 and 48 hrs. Culture supernatants were collected and filtered using 0.2 microns filter. Supernatants were stored at −80°C until analysis for cytokines, chemokines and growth factors using custom Bioplex Human Cytokine 17-plex from Bio-Rad laboratories (Hercules, CA) with detection using Luminex 100 IS system (Upstate Biotechnology, Lake Placid, NY).

## Results

### Identification of Motifs in the Cytoplasmic Tail of M-4 that Regulate Expression

**Figure 1:**
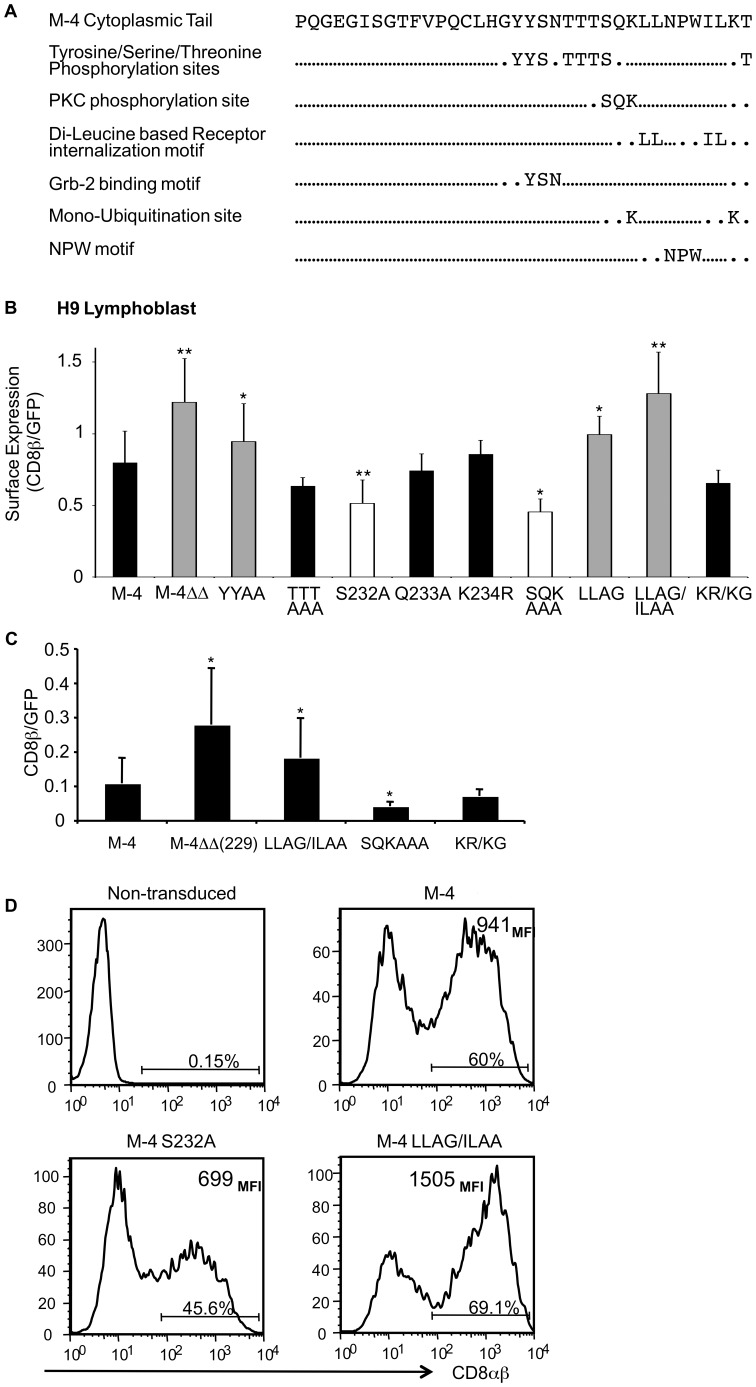
Motifs in the cytoplasmic tail of the M-4 isoform that regulate cell surface expression. (**A**) Potential amino acid motifs in the cytoplasmic tail of M-4 isoform (www.expasy.org). (**B**) Quantitative analysis of the surface expression of the M-4 wild-type and mutant proteins expressed in H9 cell line using the anti-CD8β antibody (5F2) by flow cytometry. The amount of CD8β binding was normalized to GFP expression. Each value corresponds to an average of three experiments. The standard deviation and two-population Student’s paired t-test was used to determine statistical differences of the mutants relative to the wild type M-4 isoform, indicated as one star * for p<0.05 and ** p<0.01. (**C**) Surface expression levels of M-4 wild-type and mutant proteins normalized to GFP expression in primary CD4^+^ T cells. Peripheral blood CD4^+^ T cells were stimulated with antibodies against CD3 and CD28 and transduced with lentiviruses expressing GFP and wild type or mutant M-4 proteins. On day 8 cell surface staining with CD8β antibody was analyzed by flow cytometry. The data are the mean +/− S.D. from three independent experiments. Values that are statistically different from the wild type M-4 protein are indicated as * p<0.05 and ** for p<0.01. (**D**) CD8αβ expression on CD4^+^ T cells prepared as in (C) expressing M-4 wild-type or mutants S^232^A or LL^235–6^AG/IL^240–1^AA. One representative experiment of five experiments is shown.

Since the divergence between the CD8β isoforms is in their cytoplasmic tails, we analyzed the 36 amino acid residues unique to the M-4 cytoplasmic tail for motifs that might regulate trafficking or signal transduction. Sequence analysis of the 36 unique residues in the M-4 tail revealed a number of potential motifs. These included potential tyrosine and serine/threonine phosphorylation sites, a protein kinase C binding motif (SQK), a Grb-2 binding motif (YXNX) [29] and lysines that could serve as sites for mono-ubiquitination (Figure 1A). In addition, a di-hydrophobic-based receptor internalization/sorting motif (LL) that potentially could bind to the clathrin-vesicle adaptor protein AP-2 was also observed. To investigate how these sequence motifs might regulate M-4 surface expression, we either deleted or mutated residues in the M-4 cDNA (Tables S1 and S2) and subsequently cloned each cDNA downstream of GFP in a lentiviral vector. The M-4 mutants were initially screened in the human T cell line H9 (CD4^+^,CD8^−^) and CD8ββ cell surface expression levels of the mutants were compared to the wild-type M-4 protein gating on cells with equivalent levels of GFP. This ensured that differences in surface expression of CD8β were from the phenotype of the M-4 mutants and not from differences in uptake or expression of the cDNAs in the lentiviral vector.

We identified mutations that affected CD8ββ M-4 protein cell surface expression (Figure 1B). A carboxy-terminal 15 amino acid deletion mutant M-4ΔΔ(229–243) showed increased surface expression as did a di-hydrophobic mutant LL^235,236^AG. The effect was greater with the double mutant (LL^235,236^AG/IL^240,241^AA). Mutation of the two tyrosines in the putative Grb-2 binding motif (YY^225,226^AA) showed an increase in the M-4 surface levels similar to LL^235,236^AG mutant. Mutation of the serine residue in the PKC phosphorylation motif (S^232^A) and the SQK^232–4^AAA resulted in reduced surface levels of M-4. The surface expression of other mutants was not significantly reduced relative to M-4 wild-type protein.

We further determined effects on surface expression of some of the mutants expressed in primary CD4^+^ T cells. Similar to the studies in H9 T cells, CD8ββ expression levels were determined for similar levels of GFP using the same lentiviral vector. Two mutants M-4ΔΔ(229–243) and LL^235–6^AG/IL^240–1^AA had higher CD8ββ expression levels compared with wild type CD8ββ M4 while the mutant SQK^232–4^AAA had reduced levels (Figure 1C) as we observed in the H9 T cells. No difference was observed for the K^234^R/K^242^G mutant (KRKG). We also expressed two mutants that either increased or decreased expression levels using a lentiviral vector co-expressing both CD8α ανδ β. The CD8αβ M4 LL^235–6^AG/IL^240–1^AA again consistently showed higher expression levels compared with CD8αβ M4 whereas the heterodimer with CD8β M4 S^232^A had reduced levels in five experiments.

### Sequence Motifs in M-4 Cytoplasmic Tail that Regulate Protein Downregulation

**Figure 2:**
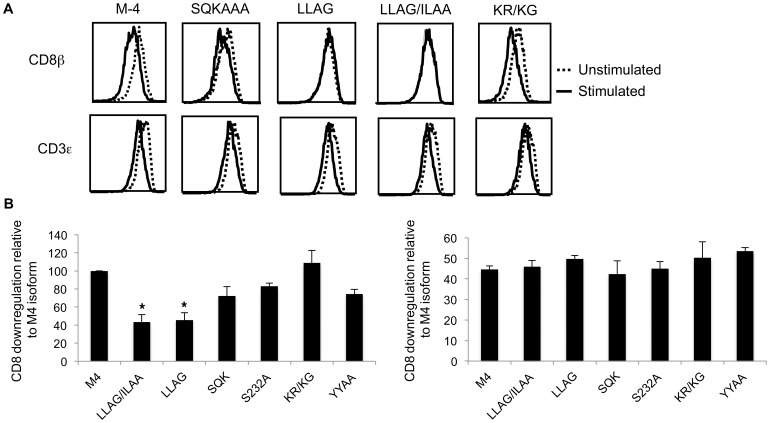
Identification of motifs signals in the cytoplasmic tail of M-4 isoform that modulate downregulation from the cell surface. (**A**) Receptor downregulation was measured by determining surface expression for CD8β M-4 wild-type and M-4 mutant levels by flow cytometry before and after stimulation of cells with PMA (100 ng/ml) for 60 minutes. One representative of 3 independent experiments is shown. (**B**) Receptor downregulation relative to CD8β M4 wild type was as in (A) quantitatively represented. Levels of CD3 protein after stimulation are indicated as well. Analyzing three experiments values that are statistically different from the wild type M-4 protein are indicated as * for p<0.05 and ** p<0.01.

The difference in protein expression levels might be a consequence of impaired downregulation rates. We checked this possibility by examining the downregulation of M-4 wild-type and a subset of mutant proteins expressed in the H9 cell line. Cells were incubated with the anti-CD8β antibodies on ice, then transferred to 37°C in medium with or without PMA for one hour. To determine cell surface levels of CD8, the cells were incubated with goat anti-mouse IgG secondary antibody conjugated to phycoerythrin, washed, and then incubated with Alexa 647 conjugated anti-CD3 antibody. The receptor downregulation relative to wild type M4 after stimulation with PMA was significantly reduced (p<0.5) for both the LL^235–6^/AG and LL^235–6^AG/IL^240–1^AA mutants suggesting a defect in downregulation (Figure 2A and 2B). The levels of CD3 were reduced on all stimulated cells but the relative CD3 downregulation was not significantly different for the different mutants (Figure 2B).

**Figure 3:**
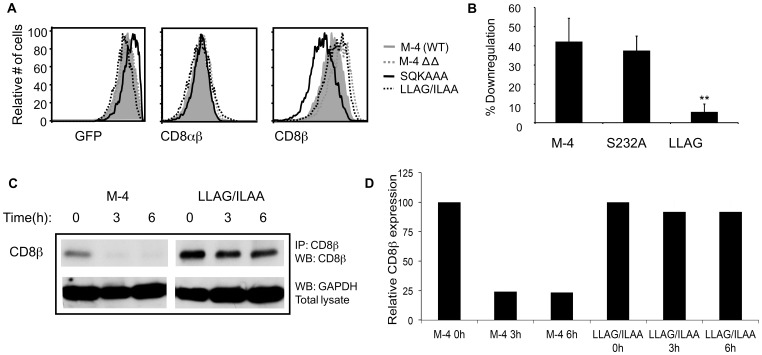
Di-leucine motif in the cytoplasmic tail of M-4 isoform regulates cell surface expression. (**A**) Histograms showing expression of GFP, CD8αβ heterodimer and total CD8β for M-4 wildtype and mutant proteins analyzed in JM thymoma by flow cytometry**.** (**B**) Percentage downregulation of total CD8β in JM expressing M-4 wild type or mutant proteins after stimulation relative to unstimulated cells. (**C**) Stability studies of CD8β M-4 wild-type and mutants. JM cells treated with cycloheximide, lysed and immunoprecipitated with anti-CD8β antibody were resolved on SDS-PAGE, transferred to a nitrocellulose membrane and probed with anti-CD8β mAb (5F2). A portion of the total cell lysate was analyzed by Western blotting with GAPDH mAb. (**D**) The CD8β protein level was determined by quantitating the band intensity for each CD8β band and plotting the mount relative to the protein amount at time zero for three independent experiments.

We also expressed mutant CD8β proteins in JM cells (CD8α^+^, ΧΔ8α^+^, CD8β^−^) with the lentiviral vector containing the GFP reporter gene. Both M-4ΔΔ (deletion 229–243) and LLAG/ILAA mutant showed a two-fold increased surface levels of total CD8β expression relative to the wild-type M-4 protein (Figure 3A) in cells expressing similar levels of GFP. In contrast, the SQK^232–4^AAA mutant showed reduced surface expression compared to the wild-type protein (Figure 3A) consistent with the results in the H9 cell line. The phenotype of the S^232^A and the LL^235,236^AG mutants were similar to the SQK^232–4^AAA and double di-hydrophobic mutants, respectively (data not shown). We then compared both CD3 and CD8 downregulation after PMA stimulation for one hour of CD8αβ M4 S^232^A and the LL^235,236^AG mutants relative to the wild type protein. Dowregulation of CD3 served as a positive control for the stimulation. We found that the LL^235,236^AG mutant showed significantly reduced receptor downregulation as compared to the M-4 wild-type and the S^232^A mutant (Figure 3B), supporting the hypothesis of a defect in downregulation of this protein. In addition, we compared the stability of the LL^235,236^AG mutant to the wild type protein after treatment with cycloheximide for 3 or 6 hours to block new protein synthesis, and found that the LL^235,236^AG mutant protein was relatively more stable (Figure 3C and 3D).

### M-4 Isoform Cytoplasmic Tail Indirectly Associates with Ubiquitinated Substrates by a Bridge Protein Binding to a Specific Motif in the Tail

**Figure 4:**
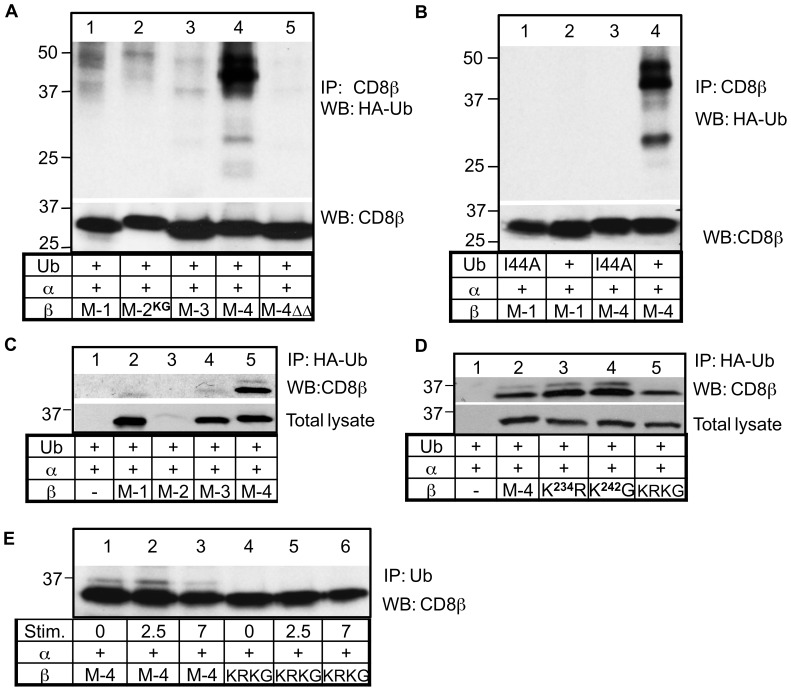
M-4 cytoplasmic tail binds to ubiquitinated proteins and is modified itself by ubiquitination. (**A**) HEK-293T cells co-transfected with plasmids expressing HA-tagged ubiquitin, CD8α and each CD8β isoform or mutant proteins. After 48 hours cells were lysed with 1% BRIJ 97 and immunoprecipitated with anti-CD8β mAb, run on a polyacrylamide gel, transferred to a membrane and probed with the anti-HA antibody. Gels were reprobed with an anti-CD8β antibody (5F2). Small differences in size reflect different lengths of the isoforms. (**B**) Co-transfection of HEK-293T cells with either wild type HA-Ub or I^44^A mutant of HA-Ub. The immunoprecipitation and Western blotting procedures were performed as in (A). (**C**) Determination of direct ubiquitination of CD8β isoforms. Cells expressing individual isoforms were immunoprecipitation with anti-HA mAb followed by blotting with anti-CD8β antibody. An aliquot of total cell lysate was similarly analyzed. (**D**) Effect of lysine mutants on M-4 ubiquitination. Cells expressing M-4 wild-type, single lysine (K^234^R, K^242^G) or double lysine (K^234^R/K^242^G labeled KRKG) mutants were lysed and protein analyzed as described in (C). (**E**) The JM T cells expressing CD8α and either CD8β M-4 or the double lysine mutant KRKG were incubated with the anti-CD8α (OKT8) and anti-CD3 (OKT3) mAbs for 0, 2.5 or 7 minutes at 37°C. Cells were lysed, immunoprecipitated with the anti-Ub mAb followed by Western blotting with the anti-CD8β antibody. A representative of three independent experiments is shown in each panel.

Mono and poly-ubiquitination of a protein is a mechanism to facilitate non-covalent interaction with proteins containing ubiquitin-binding domains (UBDs). These domains are generally small (20–150 amino acids), and independently fold. They are structurally diverse and include domains such as the ubiquitin-associated (UBA) domain, ubiquitin-interacting motif (UIM) and CUE domain (coupling of ubiquitin conjugation to endoplasmic reticulum degradation) [30]. Secondary structure prediction for the M-4 cytoplasmic tail using the PSIPRED protein structure prediction server revealed that M-4 had potential alpha helical regions that are characteristic of some UBD domains. We tested the hypothesis that the M-4 cytoplasmic tail could bind mono-ubiquitinated proteins. Plasmids expressing wild type CD8β isoforms (M-1 to M-4) or M-4 ΔΔ (229–243 deletion) mutant were individually co-transfected with human CD8α and HA-tagged ubiquitin into the HEK-293T cell line. Cells were lysed under mild conditions, CD8β immunoprecipitated and Western blotting performed with an anti-HA antibody to determine association with HA-tagged ubiquitinated targets. Several ubiquitinated species of both higher and lower molecular weight than CD8β were pulled down with the anti-CD8β antibody from cells expressing the M-4 isoform (Figure 4A, lane 4) whereas from cells expressing M-1, M-2, or M-3 isoforms there were a few weak bands (Figure 4A, lane 1–3). The M-4ΔΔ (deletion 229–243) mutant, failed to pull down the same ubiquitinated targets (Figure 4A, lane 5) indicating the critical importance of the M-4 cytoplasmic tail for the association. In addition, no bands were detected in cells expressing the CD8αα protein (data not shown). Reprobing the same membrane with an anti-CD8β antibody detected a single band corresponding to the size of the full length protein of the isoforms (Figure 4A).

To determine the specificity of the interaction, we repeated the experiment co-expressing a mutant form of HA-tagged ubiquitin (I^44^A). The alpha helices of the UBA and UIM domains interact with a hydrophobic patch on ubiquitin and I^44^ is a critical residue for this interaction [31]. The resulting mutation at the I^44^A ubiquitin showed a loss in the pull-down of ubiquitinated targets after M-4 immunoprecipitation (Figure 4B, lane 4) as compared with the wild type ubiquitin (Figure 4B, lane 3), supporting the hypothesis that a UBA domain is important for association with ubiquitinated substrates. Reprobing the same membrane with an anti-CD8β antibody detected a single band corresponding to the full length protein (Figure 4B).

**Figure 5:**
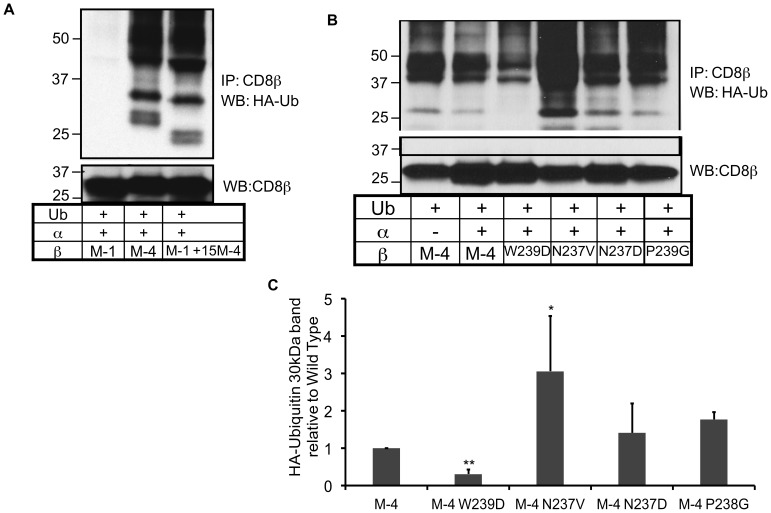
The NPW motif in the M-4 cytoplasmic tail mediates binding to ubiquitinated proteins. (**A**) HEK-293T cells co-transfected with plasmids expressing HA-tagged ubiquitin, and CD8β wild type M1, M4 or chimeric protein M1 with 15 amino acids of the C terminus of the M4 cytoplasmic tail. After 48 hrs cells were lysed with 1% BRIJ 97, precipitated with anti-CD8β mAb and run on a polyacrylamide gel. Western blotting was performed with the indicated antibody. The membrane was then stripped and re-probed with the anti-CD8β antibody. The experiment was repeated three times. (**B**) HEK-293T cells co-transfected with plasmids expressing HA-tagged ubiquitin, and CD8β wild type or mutant proteins. The immunoprecipitation and Western blotting experiments were performed as in (A). A representative of four experiments is depicted. (**C**) The intensity of the 30 kDa band was analyzed and the amount of the 30 kDa band of the wild type relative to each mutant protein is represented. The levels of CD8β protein detected after reprobing with anti-CD8β antibody were used to correct for differences in protein expression between experiments. Student’s paired t-test was used to determine statistical differences of the mutants relative to the wild type M-4 isoform, indicated as one star * for p<0.05.

The interaction with ubiquitinated substrates could also occur if there was a protein interacting with the M-4 cytoplasmic tail that itself contained a UBD domain. In this case the interaction of M-4 with ubiquitinated substrates would be indirect. We linked the last 15 amino acids (229–243) of the M-4 cytoplasmic tail to the end of the M-1 tail. We then determined whether the fusion protein could also interact with ubiquitinated substrates using the same approach as described above. The chimeric protein was able to co-immunoprecipitate ubiquitinated substrates similarly to the wild type M-4 protein except for the lower doublet that was shifted in the blot indicting a lower molecular weight (Figure 5A). This is consistent with a bridge protein interacting with the M4 cytoplasmic tail rather than the cytoplasmic tail forming a UBD which generally has 20 amino acids or more.

We therefore examined the residues in the terminal 15 amino acids that were required for the interaction with ubiquitin targets for a potential motif that might bind a protein. We noted the residues NPW that are similar to the NPF motif that associates with proteins containing an Eps15 homology (EH) domain. This domain also binds to other motifs such as FW, WW, SWG and HTF [32] indicating some degree of degeneracy. Since some EH domain-containing proteins such as Eps15, a protein involved in endocytosis, also contain a UIM domain [33], this type of protein could serve as a bridge protein. Therefore, we created four single amino acid mutations in the NPW motif and determined the effect on association between CD8β M-4 and ubiquitinated substrates. Of the four mutants, two mutants significantly affected the interaction; one mutant (N^237^V) enhanced the interaction while another mutant (W^239^D) greatly diminished the association (Figure 5B and 5C). This supports the hypothesis that these residues play a role in interaction with a protein containing a domain that can interact with mono-ubiquitinated substrates.

### Ubiquitination of the M-4 Cytoplasmic Tail

We next tested whether the lysines in the tail of M-4 isoform could be modified by ubiquitination. Cells expressing individual isoforms were lysed by boiling, which disrupts weak or hydrophobic interactions, and HA-tagged ubiquitin containing proteins were immunoprecipitated with the anti-HA antibody. A Western blot was performed and the membrane probed with the anti-CD8β antibody. For cells expressing the M-4 isoform, we observed a strong band corresponding to the CD8β protein and a higher band of ∼40kDa that is an increase in 8 kDa in size relative to unmodified CD8β (∼32 kDa). This size increase corresponds to the size of a single ubiquitin. Two lysines in the M-4 tail are potential ubiquitination sites. We therefore mutated each site individually (K^234^R or K^242^G) or together (K^234^R/K^242^G) and determined if the protein was still mono-ubiquitinated. Mutation of individual lysines retained the mono-ubiquitinated band (Figure 4D, lane 3 & 4), however, when both lysines (K^234^R/K^242^G) were mutated, the protein could no longer be modified by ubiquitination (Figure 4D, lane 5). The presence of un-ubiquitinated CD8β protein in Figure 4D was consistent with the previous finding of association of CD8β with ubiquitinated substrates. In this case we immunoprecipitated the ubiquitinated substrates and blotted with the anti-CD8β antibody, the complementary approach of the experiments in Figure 4A. These results further support the existence of this interaction.

We confirmed the monoubiquitination of the M-4 protein in the human T cell line JM that expressed CD8α and was transduced to express either wild-type CD8αβ M-4 or the mutant CD8αβ M-4 (K^234^R/K^242^G) that was defective for mono-ubiquitination in the 293T cells. Because this is a T cell line, we were able to examine mono-ubiqutination of CD8β M-4 in both unstimulated and stimulated cells by antibody crosslinking with anti-CD8α and anti-CD3 antibodies. The cells were lysed in the presence of deubiquitinating enzyme blockers, immunoprecipitated with an anti-Ub antibody and probed with the anti-CD8β antibody. We found a strong band corresponding to the size of CD8β and another band that was about 8 kDa higher that would correspond to a mono-ubiquitinated form of CD8β (Figure 4E), similar to the band seen in the 293T experiments. However, with the mutant CD8β protein lacking the two lysines, the mono-ubiquitinated form was absent clearly demonstrating that M-4 protein is ubiquitinated both in unstimulated (Figure 4E, lane 1) and stimulated cells (Figure 4E, lane 2). Interestingly, the band was more intense after 2.5 mins. of stimulation and then decreased at 7 mins. (Figure 4E, lane 3). The presence of the un-ubiquitinated CD8β band resulted from pull down of the protein incubated with the OKT8 anti-CD8α antibody in both stimulated and unstimulated cells. The unstimulated cells were treated identically but not warmed to 37°C. The presence of the un-ubiquitinated CD8β band resulted from pull down of the protein as a consequence of the presence of the OKT8 antibody used for the stimulation step. The band was absent when the OKT8 antibody was omitted (data not shown). Therefore, in both the 293T cells and the JM T cells we could detect mono-ubiquitinated CD8β M-4.

### Skewed Cytokine and Chemokine Production by JM Thymoma Expressing M-4 Isoform

To investigate the functional role of the M-4 isoform compared with the M-1 CD8β isoform we tested their ability to modulate cytokine production in a CD8 T cell line. Lentiviral vectors expressing M-1 or M-4 isoform were transduced into the JM thymoma cells (CD4^+^, CD8α^+^, CD8β^−^) to generate stable cell lines that differed only in the expression of each CD8β isoform. Cells were sorted for comparable levels of expression of CD8β and stimulated with plate bound anti-CD3 and anti-CD8 antibody to determine the cytokine profile of these cell lines. Cells expressing the M-1 isoform showed significantly higher levels of IFN-γ while cells expressing the M-4 showed 3-fold higher MIP-1β (Figure S2). This latter observation is consistent with our previous finding of the predominant expression of M-4 isoform in terminally differentiated effector memory CD8 T cells [22] and a study showing skewing of late effector memory CD8^+^ T cells towards MIP-1β chemokine production and decreased IL-2 production [24].

### The Frequency of MIP-1β Producing Primary Human T Cells Expressing M-4 Isoform was Higher After Antigen Stimulation

**Figure 6:**
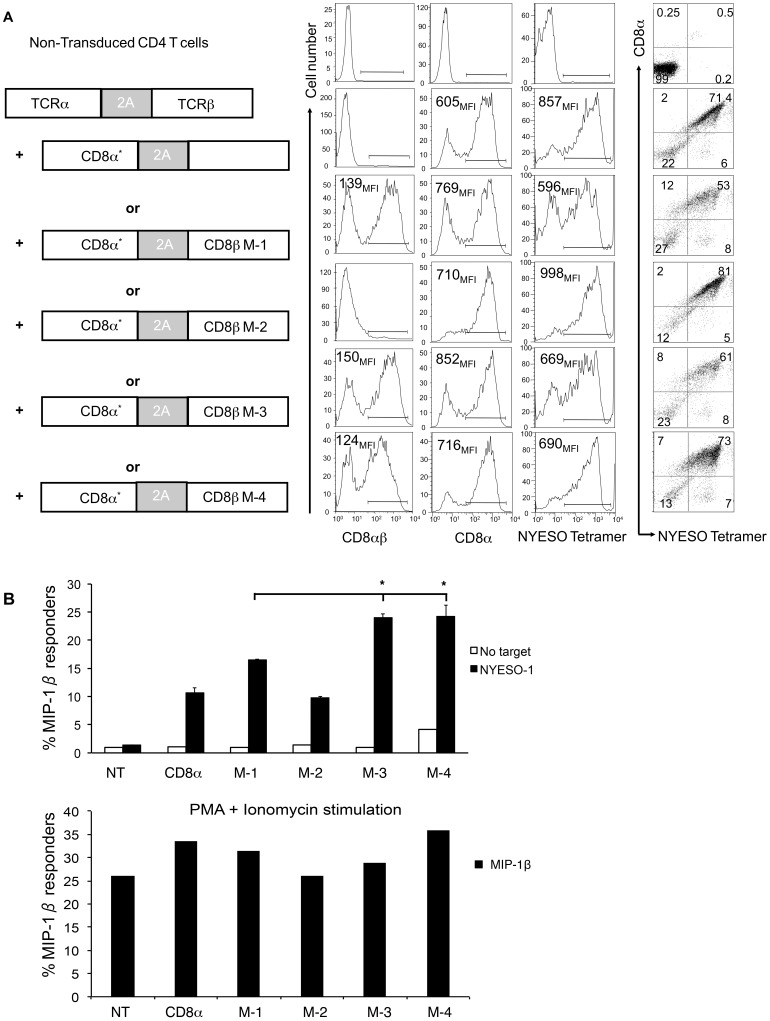
CD4^+^ T cells transduced with the M-4 CD8β isoform showed increased frequency of cells producing MIP-1β after stimulation. Peripheral blood CD4^+^ T cells were stimulated with anti-CD3 and anti-CD28 antibodies for 24 hours, and then co-transduced with a lentivirus expressing a NY-ESO-1 TCR and another lentivirus expressing CD8α and one of the CD8β isoforms. Cells were stimulated and analyzed for cytokine/chemokine production after day 10–12. (**A**) Schematic representation of lentiviral vectors used for co-transduction of primary CD4^+^ T cells is followed by histograms for surface expression of CD8αβ, CD8α and TCR and dot plots showing cell population co-expressing NY-ESO-1 TCR and CD8α. Data were collected by flow cytometry using antibodies against CD8 and an MHC tetramer specific to NY-ESO-1 (NY-ESO- tetramer). Live CD3^+^ lymphocytes were gated using side vs. forward scatter, anti-CD3 antibody and live/dead cell dye. (**B**) Frequency of transduced CD4^+^ T cells producing MIP-1β (top panel) after stimulation with K562 target cells expressing the NY-ESO-1 antigen. T cells without targets served as negative control and cells stimulated with PMA and Ionomycin (bottom panel) were used as positive control. One representative of three independent experiments is shown. Values that are statistically different from the wild type M-1 protein as determined by two-population Student’s paired t-test are indicated as one star (*) for p<0.05.

To further determine whether the M-4 isoform could contribute to skewing T cells towards MIP-1β production, we tested a more physiological peptide-specific system using normal peripheral blood lymphocytes. We co-transduced primary CD4^+^ T cells with individual isoforms and an MHC class I restricted TCR. Using CD4^+^ T cells eliminated the concern of endogenous CD8 protein present in CD8^+^ T cells. Purified CD4^+^ T cells were stimulated with beads coated with anti-CD3 and anti-CD28 antibodies and co-transduced with a lentivirus expressing codon-optimized CD8α and individual CD8β isoforms and a second lentivirus expressing TCRαβ from a CD8 dependent TCR recognizing the melanoma antigen NY-ESO-1 (Figure 6A) [34]. This TCR binds to HLA-A2 and to one of the most immunogenic HLA-A2-restricted peptides NY-ESO-1^157–165^ [35]. Expression levels of CD8 were monitored with antibodies that bind to CD8α (OKT8) or to CD8αβ (2ST8) and for TCR expression we used a NY-ESO-1 HLA-A2 tetramer specific for NY-ESO-1 TCR (Figure 6A). Experiments were performed when at least 50% of the cells expressed similar levels of CD8 and TCR on cells expressing M-1, M-3 and M-4 isoforms; in some cases we corrected for differences in % positive by mixing cells expressing each isoform with non-transduced cells so that the number of TCR positive cells was similar for each sample. The M-2 protein was not expressed on the cell surface consistent with our previous finding that the protein is degraded in resting cells [22]. After reaching a resting state on day 10–12, transduced CD4^+^ T cells were re-stimulated with K562 (myelogenous leukemia line) target cells that were modified to express HLA-A2, GFP, and the NY-ESO-1 antigen [36]. An intracellular cytokine assay was performed after stimulating the cells and testing for cytokine secretion (MIP-1β, IL-2, and IFN-γ).

We found a significantly higher percentage of T cells producing MIP-1β when T cells expressed either the CD8β M-3 or M-4 isoform after stimulation with HLA-A2^+^ K562 cells expressing NY-ESO-1 (Figure 6B). This statistical significance was found when we compared the response of cells expressing M-3 and M-4 with either cells expressing CD8α alone or CD8β M-1. No response was observed with non-transduced CD4^+^ T cells (Figure 6B). Cells expressing CD8α alone or the M-2 isoform had about half the frequency of responders compared with their frequency in the M-3 and M-4 isoforms despite the fact that TCR expression levels were higher for cells expressing CD8α or M-2. The quantitative differences observed for secretion of IL-2 and IFNγ were too minimal to be interpreted (data not shown). These results suggest that cells expressing CD8 co-receptor with either the CD8β M-4 or M-3 isoform has the potential to enhance the quality of TCR recognition for pMHCI relative to the M-1 isoform with respect to the frequency of MIP-1β producing cells.

## Discussion

When a gene acquires additional exons during evolution, such as likely occurred in the ancestor to humans and chimpanzees, initially there is minimal splicing to these exons. [37] However, if the new proteins that arise have a selective advantage, inclusion of the new exons in the spliced product increases during evolution. Such is the case of human CD8β isoform M-4 that is predominantly expressed in effector memory CD8T cells and arises from a spliced mRNA that includes information from the new exons added to the human/chimpanzee ancestral CD8B gene.

For long-lived species such as humans, memory T cells are an important subset contributing to success in fighting off previously seen pathogens. In this study, we demonstrate that the human CD8β isoform M-4 has a 36 amino acid cytoplasmic tail that confers unique properties to the CD8 coreceptor function that distinguishes it from the M-1 isoform, the dominant form in naive T cells and the murine homologue. We found that the di-hydrophobic residues in the M-4 cytoplasmic tail were important in regulating cell surface expression and downregulation. An NPW motif was important for association with ubiquitinated substrates and the M-4 protein itself could be mono-ubiquitinated on either of two lysine residues. Lastly, we found enhanced responsiveness towards MIP-1β production in peripheral blood CD4^+^ T cells with an MHC class-I restricted TCR which is consistent with polarization of late memory CD8 T cells towards MIP-1β chemokine production [24].

Interestingly, effector memory T cells express a relatively lower level of CD8 on the cell surface as compared with central memory or naïve T cells. One possible effect of reduced CD8 levels would be to cause memory T cells to respond to cells expressing a specific peptide-MHCI while limiting response to cells expressing cross-reactive peptide-MHCI complexes with lower affinity. CD8 influences the degree of T cell cross-reactivity as the presence of CD8 extended the range of peptide-MHC ligands recognized by a given TCR [38]. Modulating levels of CD8 by increasing CD8 expression levels increased responsiveness to low-affinity TCR ligands and lowering the level of CD8 reduced the ability of the T cells to respond to lower avidity peptide-MHC [39]. Thus, keeping CD8 expression low could contribute to lowering cross-reactivity and modulating the size and responsiveness of an antigen-specific T cell pool. The presence of the dihydrophobic motif (LL^235^) in the M-4 isoform cytoplasmic tail could contribute to regulating cell surface levels of the CD8 co-receptor in the effector memory T cells. Changes in rates of receptor downregulation and/or trafficking for recycling or degradation could modulate cell surface receptor expression levels.

The ability of the di-hydrophobic motifs to regulate cell surface expression of CD8 coreceptor could occur because of interaction with the clathrin adaptor protein AP-2 [40]. One consensus sequence for binding of endocytic di-leucine motifs by the AP-2 complex is [ED]xxxL[LI]. This motif is found in the CD3γ chain (DxxxLL) that is responsible for binding AP-1 and AP-2, and linking the TCR to the clathrin-dependent internalization [41]. While the acidic residues are normally glutamate or aspartate, other residues with the potential for a negative charge are also possible. For instance, the human and feline CD4 protein binds to AP-2 through a di-leucine based motif and the upstream residues providing a negative charge are either glutamine or histidine respectively [42]. Alternatively, a phosphorylated serine can substitute for a negatively charged residue and the charged residue can be at −3 or −5 position as well [26]. In the case of M-4, at site -3 from the LL residues there is a serine residue preceded by a tandem threonine stretch (TTTSQKLL) that potentially could be phosphorylated. The inability of M-4 dihydrophobic motif mutants to associate with the AP2 complex could explain the decreased downregulation and increased surface expression. Interestingly, neither the human CD8α chain nor CD8β M-1 isoform has such an endocytic motif.

The other motif that could affect trafficking of the CD8 coreceptor is serine at position 232, a potential PKC phosphorylation site, in M-4 tail. The PKC motif SQK^232–4^ regulates expression by affecting endocytic sorting [43], [44]. The serine mutation led to less expression on the cell surface consistent with a possible effect on sorting and trafficking.

The ability of the M-4 protein to associate with ubiquitinated proteins and to be monoubiquitinated may also affect trafficking or potential interaction with adaptor proteins. A number of proteins in the endocytic pathway have ubiquitin binding domains [33] and affect receptor trafficking. For instance, mono-ubiquitination of Igβ was required for proper endosomal sorting and presentation of antigen to T cells but not for receptor internalization [45]. The CD8β M-4 protein also associated with ubiquitinated proteins and the NPW motif influenced the interaction. Because EH domain proteins bind to NPF it is possible that such a protein with a UBD domain as well serves as a bridge protein linking M-4 to ubiquitinated substrates. One candidate protein is Eps15 [33]. The bridge protein may facilitate interaction with proteins in the endocytic pathway or interaction with CD3ζ, CD3δ, and/or ZAP-70 that are mono-ubiquitinated on lysines after activation by the E3 ubiquitin ligase Cbl-b [46], [47]. In addition, mono- and polyubiquitination occurs on the critical adapter protein LAT, a scaffold protein that docks many Src homology 2 (SH2) proteins [48] and is known to interact with CD8 [13]. These interactions could impact the dwell time of receptors on the plasma membrane versus in early endosomes and therefore affect downstream signal transduction cascades. [49], [50].

The rate-limiting step in T cell:APC interaction is the formation of an immunological synapse following which, T cell activation and receptor signaling occurs. The secretion of cytokines such as MIP-1β by CD8 effector memory T cells occurs as a consequence of signaling through CD8 and the TCR along with a co-stimulatory signal. The influence of the M-4 isoform on CD8 coreceptor function in modulating cytokine production by T cells is supported by our finding that there was 2-fold more MIP-1β producing T cells after activation of cells expressing the M-4 isoform as compared with the M-1 isoform. The input signals received by CD8^+^ T cells expressing the M-4 isoform may both qualitatively and/or quantitatively fine-tune the activation threshold.

Understanding the significance and function of human CD8β isoforms, specifically M-4 will be useful in determining the role of CD8 coreceptor in fine tuning the function and/or survival outcome of human memory cells. These findings could also be useful for development of redirected T cells for immunotherapy against cancer cells. Modification of cytotoxic CD8^+^ T cells to enhance anti-tumor efficacy, survival and expansion in the host is an area of active investigation. For instance, chimeric antigen receptors (CARs) with intracytoplasmic domains from proteins such as TCRζ and costimulatory proteins were added to autologous T cells [51]. Understanding the role of motifs in the cytoplasmic tails of cell surface proteins important in CD8^+^ T cells could potentially aid in the design of optimal chimeric proteins for fine-tuning of T cell responses.

## Supporting Information

Figure S1 **Schematic representation of the alternatively spliced isoforms of the human CD8B gene.** The unique amino acid sequences of the cytoplasmic tail of each isoform (M-1, M-2, M-3 and M-4) are shown in gray boxes.
(TIF)

Figure S2 **JM thymoma T cells transduced with the either the M-1 or M-4 CD8β isoform produced a different pattern of cytokines after stimulation by antibody crosslinking.** The JM T cell thymoma cells (CD4^+^, CD8α^+^,CD8β^−^), M-1 and M-4 transfectants were stimulated with plate bound anti-CD3 and anti-CD28 for 48 hrs. The supernantants were collected, filtered and analyzed for cytokine/chemokines with a Bioplex human cytokine 17-plex using the Luminex 100 IS system. The results from one of three experiments is shown. The star indicates significant difference (p<0.05) from the JM line using the t-test.
(TIF)

Table S1 **Sequence of the Primers used for PCR amplification and cloning’s.**
(DOCX)

Table S2 **Summary of the M-4 cytoplasmic tail mutants showing the change in surface expression levels and rate of internalization (Int.) relative to the wild-type.** M-4 mutants that showed a change in surface expression are highlighted in bold. Surface expression of wild-type M-4 protein is indicated by ++ sign; ++++ represents increase and + represents decrease in surface expression relative to the wild-type. In addition, the rate of internalization is shown as no change (−); slow or not-determined (n.d.).
(DOCX)
