# Supplementary figures and images for: The Human CD8β M-4 Isoform Dominant in Effector Memory T Cells Has Distinct Cytoplasmic Motifs That Confer Unique Properties
Source: PLoS One. 2013 Mar 22;8(3):e59374. doi: 10.1371/journal.pone.0059374 (PMC3606432; doi:10.1371/journal.pone.0059374)

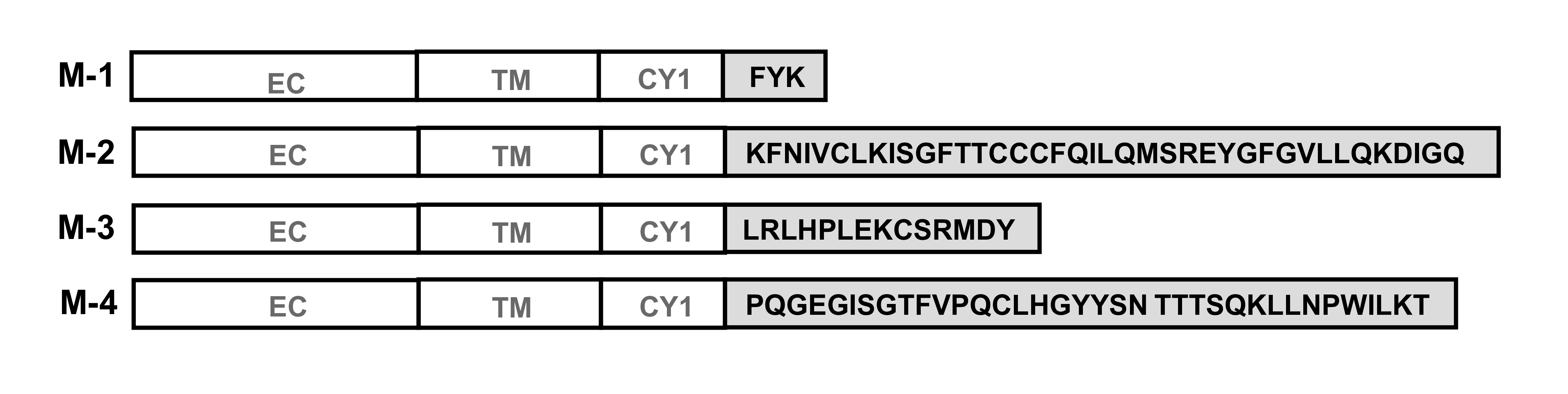

Supplement: Figure S1 — Schematic representation of the alternatively spliced isoforms of the human CD8B gene. The unique amino acid sequences of the cytoplasmic tail of each isoform (M-1, M-2, M-3 and M-4) are shown in gray boxes. (TIF) [file pone.0059374.s001.tif]

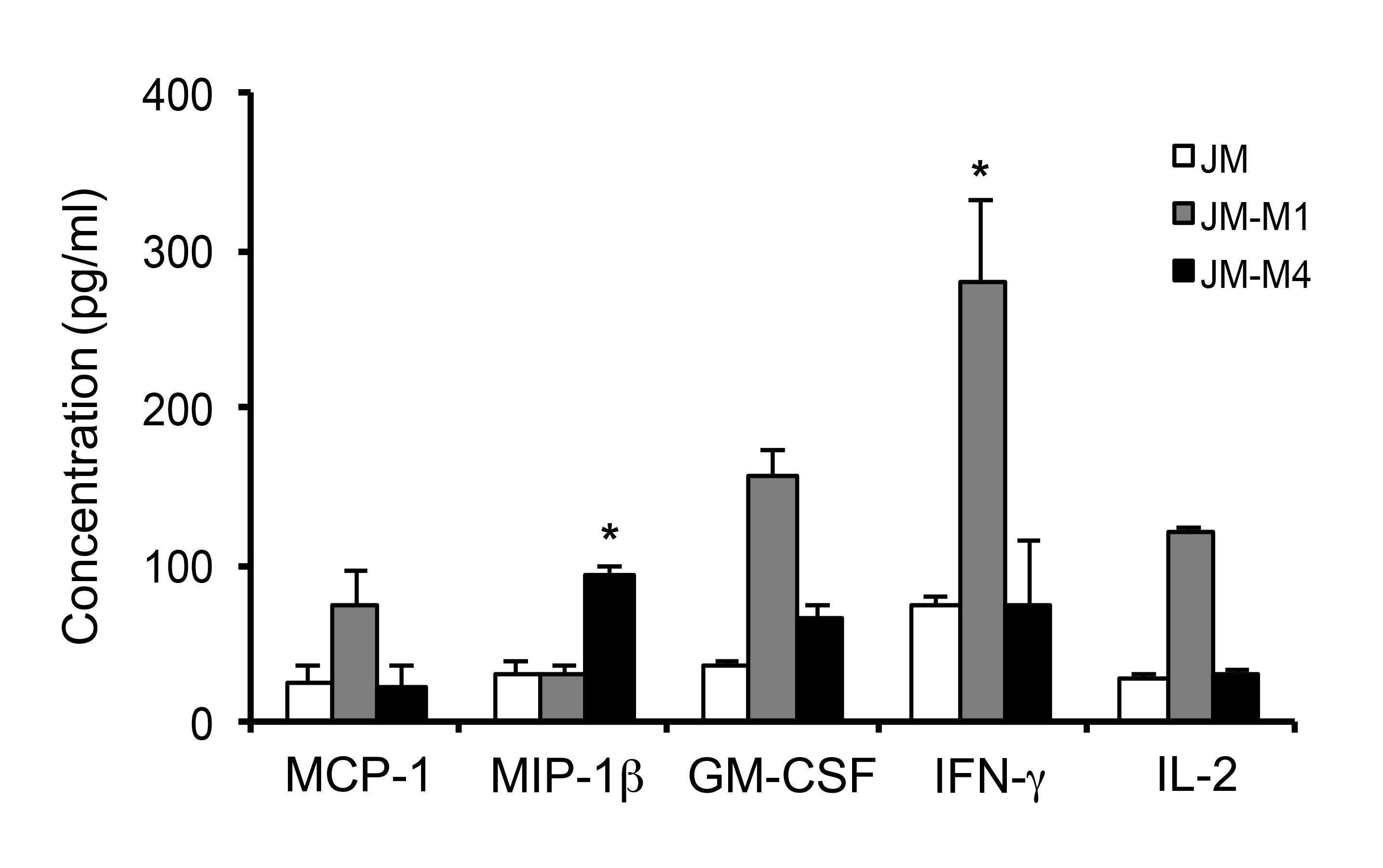

Supplement: Figure S2 — JM thymoma T cells transduced with the either the M-1 or M-4 CD8β isoform produced a different pattern of cytokines after stimulation by antibody crosslinking. The JM T cell thymoma cells (CD4+, CD8α+,CD8β−), M-1 and M-4 transfectants were stimulated with plate bound anti-CD3 and anti-CD28 for 48 hrs. The supernantants were collected, filtered and analyzed for cytokine/chemokines with a Bioplex human cytokine 17-plex using the Luminex 100 IS system. The results from one of three experiments is shown. The star indicates significant difference (p<0.05) from the JM line using the t-test. (TIF) [file pone.0059374.s002.tif]
