# Supplementary material for: The Human CD8β M-4 Isoform Dominant in Effector Memory T Cells Has Distinct Cytoplasmic Motifs That Confer Unique Properties
Source: PLoS One. 2013 Mar 22;8(3):e59374. doi: 10.1371/journal.pone.0059374 (PMC3606432; doi:10.1371/journal.pone.0059374)
Supplement: Table S1 — Sequence of the Primers used for PCR amplification and cloning’s. (DOCX) [file pone.0059374.s003.docx]

**Supplementary Table I:** Sequence of the Primers used for PCR amplification and cloning’s.

| **Primers** | **Cloning into Lentiviral vector pELNS** |
| --- | --- |
| Common Forward | GTACCTAGGATGCGGCCGCGGCTGT |
| Reverse primer M-1 | GCCGGTCGACTCATTTGTAAAATTGTTTCATG |
| Reverse primer M-2 | GGTTGTCGACTTACTGACCGATGTCTTTTTGTAGCAGGAC |
| Reverse primer M-3 | GCCGGTCGACTCAGTAGTCCATTCTGGAACATTTC |
| Reverse primer M-4 | GCTGGTCGACCTATGTTTTCAGGATCCATGGGTT |
|  |  |
| **M-4 Mutants** | **Mutagenesis Primers** |
| YYAA | CCAATGCCTGCATGGAGCAGCCAGCAATACTACAACC |
| TTTAAA | TGGATACTACAGCAATGCGGCCGCCTCACAGAAGCTGCT |
| S232A | CAGCAATACTACAACCGCTCAGAAGCTGCTTAACC |
| Q233A | CAATACTACAACCTCAGCGAAGCTGCTTAACCCA |
| K234R | ACTACAACCTCACAGAGGCTGCTTAACCCATGG |
| SQKAAA | CAGCAATACTACAACCGCGGCCGCGCTGCTTAACCCATGG |
| LLAG | ACAACCTCACAGAAGGCCGGCAACCCATGGATCCTG |
| ILAA | GAAGCTGCTTAACCCTTGGGCCGCGAAAACATAGGCAAGAAG |
| LLAG/ILAA | CTACAACCTCACAGAAGGCCGGCAACCCTTGGGCCGCG |
| K242G | AACCCATGGATCCTGGGTACCTAGGCAAGAAGCACA |
| KKRG | ACTACAACCTCACAGAGGCTGCTTAACCCATGG |
|  | **M-4 Deletion Mutants** |
| Common Forward | ACGAATTCCGGGCGCGCCACGATGC |
| M-4∆(237-243) | GGATCTAGAGCTAAAGCAGCTTCTGTGA |
| M-4∆∆(229-243) | GGATCTAGACTAATTGCTGTAGTATC |
