# Supplementary material for: The Human CD8β M-4 Isoform Dominant in Effector Memory T Cells Has Distinct Cytoplasmic Motifs That Confer Unique Properties
Source: PLoS One. 2013 Mar 22;8(3):e59374. doi: 10.1371/journal.pone.0059374 (PMC3606432; doi:10.1371/journal.pone.0059374)
Supplement: Table S2 — Summary of the M-4 cytoplasmic tail mutants showing the change in surface expression levels and rate of internalization (Int.) relative to the wild-type. M-4 mutants that showed a change in surface expression are highlighted in bold. Surface expression of wild-type M-4 protein is indicated by ++ sign; ++++ represents increase and + represents decrease in surface expression relative to the wild-type. In addition, the rate of internalization is shown as no change (−); slow or not-determined (n.d.). (DOCX) [file pone.0059374.s004.docx]

**Supplementary Table II:** Summary of the M-4 cytoplasmic tail mutants showing the change in surface expression levels and rate of internalization (Int.) relative to the wild-type.

| **Mutants** | **Cytoplasmic Tail Sequence (208-243 aa)** | **Levels** | **Int.** |
| --- | --- | --- | --- |
| M-4 W.T. | PQGEGISGTFVPQCLHGYYSNTTTSQKLLNPWILKT | ++ | - |
| **M-4∆(237)** | PQGEGISGTFVPQCLHGYYSNTTTSQKLL | ++++ | n.d. |
| **M-4∆∆(229)** | PQGEGISGTFVPQCLHGYYSN | ++++ | slow |
| **YYAA** | ……………………………..AA…………………………. | ++++ | - |
| TTTAAA | …………………………………..AAA…...……………… | ++ | - |
| **S232A** | ……………………………………….A………………….. | + | - |
| Q233A | …………………………………………A………………... | ++ | n.d. |
| K234R | …………………………………………..R………………. | ++ | n.d. |
| **SQKAAA** | …………………………………………AAA……………. | + | - |
| **LLAG** | …………………………………………...AG……………. | ++++ | slow |
| ILAA | ……………………………………………………….AA.. | n.d. | n.d. |
| **LLAG/ILAA** | …………………………………………...AG………AA.. | ++++ | slow |
| K242G | …………………………………………………………..G. | n.d. | - |
| K234R/  K242G | …………………………………………..R…………….G. | ++ | - |
